# An Accurate and Robust Method for Absolute Pose Estimation with UAV Using RANSAC

**DOI:** 10.3390/s22155925

**Published:** 2022-08-08

**Authors:** Kai Guo, Hu Ye, Xin Gao, Honglin Chen

**Affiliations:** Northwest Institute of Nuclear Technology, Xi’an 710024, China

**Keywords:** pose estimation, RANSAC, perspective-n-point, UAV, high outlier ratio

## Abstract

In this paper, we proposed an accurate and robust method for absolute pose estimation with UAV (unmanned aerial vehicle) using RANSAC (random sample consensus). Because the artificial 3D control points with high accuracy are time-consuming and the small point set may lead low measuring accuracy, we designed a customized UAV to efficiently obtain mass 3D points. A light source was mounted on the UAV and used as a 3D point. The position of the 3D point was given by RTK (real-time kinematic) mounted on the UAV, and the position of the corresponding 2D point was given by feature extraction. The 2D–3D point correspondences exhibited some outliers because of the failure of feature extraction, the error of RTK, and wrong matches. Hence, RANSAC was used to remove the outliers and obtain the coarse pose. Then, we proposed a method to refine the coarse pose, whose procedure was formulated as the optimization of a cost function about the reprojection error based on the error transferring model and gradient descent to refine it. Before that, normalization was given for all the valid 2D–3D point correspondences to improve the estimation accuracy. In addition, we manufactured a prototype of a UAV with RTK and light source to obtain mass 2D–3D point correspondences for real images. Lastly, we provided a thorough test using synthetic data and real images, compared with several state-of-the-art perspective-n-point solvers. Experimental results showed that, even with a high outlier ratio, our proposed method had better performance in terms of numerical stability, noise sensitivity, and computational speed.

## 1. Introduction

Estimating the camera pose from 2D–3D point correspondences is a key step in computer vision [1,2,3,4], SLAM (simultaneous localization and mapping) [5,6,7], and photogrammetry [8,9], via a process called camera pose estimation [10,11]. Many methods have been proposed to solve this problem with different numbers of 2D–3D point correspondences to work with different cases, which are named PnP (perspective-n-point problem) solvers [12,13,14,15,16,17]. The camera pose contains six parameters, i.e., three rotation parameters and three translation parameters. If the pose parameters are all unknown and the intrinsic camera parameters are all known, the minimal subset for pose estimation only contains three 2D–3D point correspondences since one point correspondence can give two constraints, and the corresponding methods are called P3P (perspective-three-point) solvers [18,19]. In addition, some methods can estimate the pose and partial intrinsic camera parameters when more than three 2D–3D point correspondences can be given. Some methods can work well with cases where the focal length is unknown, and the size of the minimal subset is four, which are called P4Pf solvers [8,20,21,22]. Actually, four 2D–3D point correspondences give eight constraints; hence, some methods can work well with cases where the focal length and radial distortion are unknown, which are called P4Pfr solvers [23,24]. The P4Pfr solvers only obtain one radial distortion parameter. If we have five 2D–3D point correspondences, three radial distortion parameters can be obtained, and the corresponding methods are called P5Pfr solvers [25,26]. If the number of point correspondences is at least six, all the intrinsic camera parameters and poses can be obtained using DLT (direct linear transform) solvers [27,28,29].

These above-described methods work well with cases where the six pose parameters are all unknown. In contrast, if the partial pose parameters are known, some methods can solve the problem using only two 2D–3D point correspondences for the minimal subset. When the vertical direction is known through IMUs (inertial measurement units), some methods can work well with cases using two 2D–3D point correspondences [30,31,32] or cases where some partial intrinsic camera parameters are unknown using more point correspondences [33,34].

We proposed a new method to estimate pose using two 2D–3D point correspondences when the camera position, but not the vertical direction is obtained [35]. In that paper, we mentioned that the proposed method would be used with RANSAC (random sample consensus) [2,36,37,38] in a future study, as currently presented herein.

Many PnP + RANSAC methods [13,39,40,41,42,43] have been proposed and work well with several point correspondences with outliers, especially when the outlier ratio is high. The mass correspondence set used in these methods is generally obtained from feature extraction if there are many feature points in the field of view (FOV) [39,44,45,46]. However, these correspondences are 2D–3D point correspondences and can only obtain the relative pose in multi-view geometry [4,15], not the absolute pose [35]. If we want to estimate the absolute pose with outliers using RANSAC, the mass 2D–3D point correspondences are needed, but it is a challenge to obtain so many correspondences in real scenarios because the position of the 3D point is hard to measure. In addition, in some cases, mass 3D points may not exist in the FOV, such as in missile testing, where the environment is mostly the desert and the FOV is mostly the sky, which hinders the obtention of mass feature points [35]. Some methods [14] use artificial 3D control points and obtain their positions using the total station or RTK (real-time kinematic), and then 2D–3D point correspondences without outliers can be given. In these cases, the number of 2D–3D point correspondences is small and can be used to estimate the pose directly by applying PnP solvers without RANSAC. However, the problem is that artificial 3D control points with high accuracy are time-consuming, and the small point set may lead low measuring accuracy.

Many cases can be solved using ground control points, either manually or naturally. However, in some cases, the layout and the acquisition of enough ground control points are challenging. For example, in missile experiments, which are mainly conducted in the desert, there are no natural feature points, and we can only deploy artificial control points. However, affected by the wind and the flow of sand, extensive work is needed to obtain enough control points and prevent their movement. This leads to a rapid increase in cost. In addition, the field of view is sometimes completely in the air. In this case, artificial control points and natural feature points cannot be established. Estimating the camera pose at this time is a difficult job, which serves as the motivation for this study. We adopt the method of a UAV mounting control point (light source) to solve the above problems. Accordingly, in this paper, we design a customized UAV (unmanned aerial vehicle) to efficiently obtain mass 3D points.

A light source and RTK are mounted on the UAV, and they share power with the UAV. The light source is used as 3D point that can be easily seen and extracted as a 2D point in the image. The RTK is used to obtain the 3D position of the light source. Actually, the RTK is not mounted on the light source; hence, the value of RTK does not perfectly agree with the 3D position of the light source. However, because the RTK and light source are both fixed, their relative position is known; consequently, we can use the relative position to obtain the position of light source as 3D point with RTK. Then the valid 2D–3D point correspondence is given. Lastly, the UAV flies in the FOV of the camera to obtain the mass 2D–3D point correspondences.

In practice, we can find that the 2D–3D point correspondences from UAV contain outliers, and the outliers consist of three parts. First, when the UAV is flying, the accuracy of the RTK mounted on it may decrease, which will bring some outliers of 3D points. Secondly, the extraction of the light source from the corresponding image may fail, which will bring some outliers of 2D points. Thirdly, wrong matches for 2D points and 3D points may occur [44]. Hence, in this paper, we use the method proposed in [35] with RANSAC to remove the outliers, which makes our method robust and obtains coarse pose estimation. Lastly, we develop a cost function in reprojection error to refine the coarse pose estimation through gradient descent, which makes our method accurate.

The remainder of this paper is organized as follows: Section 2 presents the problem and method statement with UAV using RANSAC, including the design of a customized UAV for point correspondence and our proposed method for pose estimation. Section 3 thoroughly tests our method in terms of robustness to camera position noise, numerical stability, performance analysis of outlier ratio, noise sensitivity, and computational speed, as well as compares it with several existing state-of-the-art PnP solvers. Section 4 provides a discussion, and Section 5 presents the conclusions.

## 2. Problem and Method Statement with UAV Using RANSAC

In this section, we design a customized UAV to obtain mass 2D–3D point correspondences, and then we propose our method using RANSAC to obtain the coarse pose. In computer vision, RANSAC is widely used to remove outliers, such as in essential matrix estimation [47,48,49]. Lastly, a cost function in reprojection error is developed to refine the coarse pose estimation on the basis of gradient descent.

### 2.1. Design of UAV for Point Correspondence

**Figure 1:**
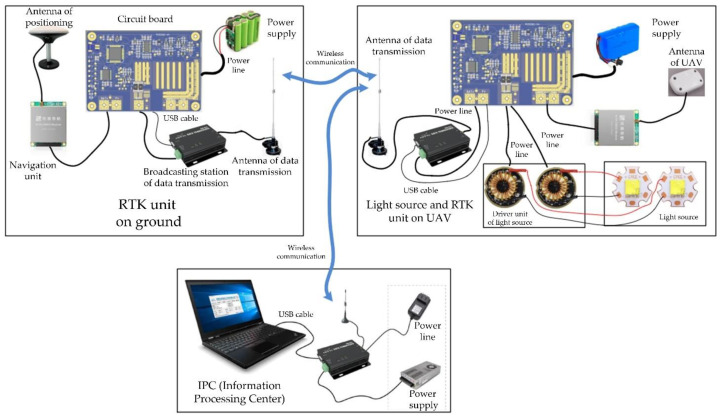
The design of the light source and RTK, exhibiting the RTK unit on the ground, the light source and RTK unit on the UAV, and the IPC (information processing center).

It is a challenge to estimate the absolute pose in cases where no feature points and buildings exist, or where the FOV is mostly the sky (e.g., missile testing in the desert). The difficulty lies in accurately placing and measuring the 3D points. In this section, we design a customized UAV with a mounted light source to obtain 3D points. In addition, an RTK is mounted on the UAV to accurately obtain the position of the light source, as shown in Figure 1.

As shown in Figure 1, the power supply on the UAV powers the light source, which is used as the correspondence point. The antenna of the UAV determines the 3D position of light source, and then the data transmission antenna transmits the data to the RTK unit on the ground and the IPC (information processing center) through wireless communication. The IPC sends instructions to the camera to obtain the 2D image and 3D position of the light source to finish the 2D–3D point correspondence acquisition.

Then, the UAV flies in the FOV of camera, and the corresponding images are simultaneously captured by the camera. The 3D position of the light source on the UAV is given by the RTK, and the 2D position of the light source in the image is obtained through feature extraction. Consequently, we can easily obtain mass 2D–3D point correspondences via correspondence mapping.

### 2.2. Pose Estimation

In Section 2.1, mass 2D–3D point correspondences were obtained through the UAV. However, they contain outliers, consisting of three parts as described in Section 1. Hence, our proposed method using RANSAC is applied in this case to obtain the coarse pose. Then, a cost function in reprojection error is developed to refine the coarse pose estimation using gradient descent.

#### 2.2.1. Pose Estimation with UAV Using RANSAC

In this paper, the camera is fixed. If all the 2D–3D point correspondences are valid, the DLT can be directly used to estimate the absolute camera pose. However, some point correspondences would be contaminated and become outliers, as described above. In this paper, two 2D–3D point correspondences are randomly selected as the minimal subset, and our proposed method [35] is used to estimate the pose. Then, the image reprojection of the 3D point can be achieved, and a cost function, $d=\left\|x_{i}-x_{i}^{’}\right\|$, is developed. Here, $x_{i}$ is the 2D point through feature extraction, and $x_{i}^{’}$ is the 2D reprojection of the 3D point $X_{i}$ after pose estimation using our proposed method. *d* is the reprojection error. RANSAC decides if the 2D–3D point correspondence ($x_{i}$ and $X_{i}$) is an inlier or outlier as follows:

(1) $$\left\{\begin{array}{cc} inlier & if\;d\leq t\\ outlier & if\;d>t \end{array}\right.,$$

where *t* is the threshold of the reprojection error, which is generally less than one pixel. Then, the number of inliers is denoted as *S*, while the number of iterations is denoted as *N*. If *S* or *N* is more than their corresponding threshold, the iteration stops; otherwise, it continues.

**Figure 2:**
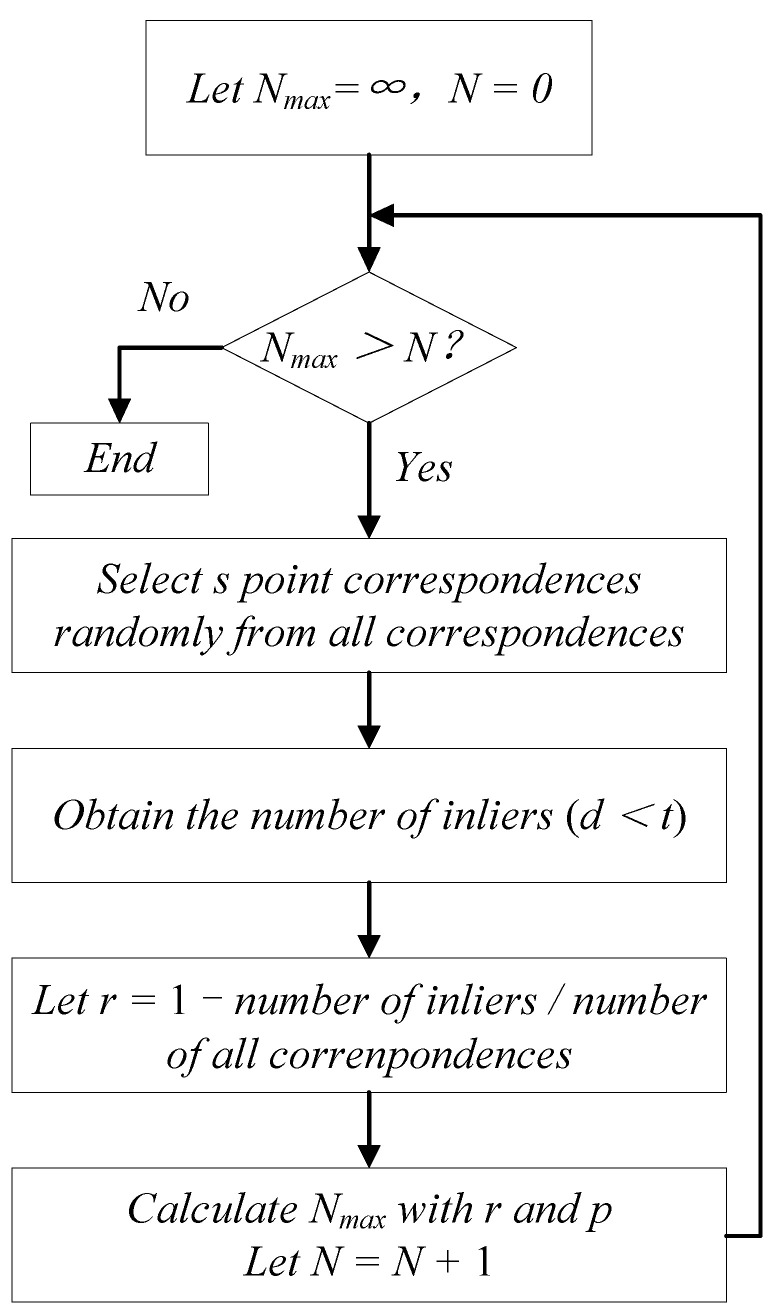
Process flow of RANSAC.

The threshold *N*_max_ of *N* is calculated using the following Equation [35]:

(2) $$N_{\max}=\frac{\log\left(1-p\right)}{\log\left(1-{\left(1-r\right)}^{s}\right)},$$

where *p* is the probability of at least one success without outliers (in this paper, we set *p* = 0.99), *r* is the outlier ratio, and *s* is the size of the minimal subset for estimation algorithm (in this paper, *s* = 2) [35]. If the UAV gives *n* 2D–3D point correspondences consisting of inliers and outliers, and the outlier ratio *r* is unknown, the adaptive algorithm for *N_max_* in RANSAC is as illustrated in Figure 2.

After the application of RANSAC, the outliers are removed, and the maximal subset of inliers is obtained, which is subsequently used for absolute pose estimation and refining.

#### 2.2.2. Normalization for Inliers

Because there are many 2D–3D point correspondences of the inliers, normalization should be applied to all 2D–3D point correspondences to improve the estimation accuracy before absolute pose estimation and refining, as described below [4]. In this paper, all 2D points were in the same frame, i.e., the image plane, and the origin was the center of image. All 3D points were in the world frame. Because we only used one UAV as the 3D point and, consequently, only one UAV was in each image, only one 2D point could be extracted from the same image.

The centroids of 2D points $\left(x_{i},1\leq i\leq n\right)$ and 3D points $\left(X_{i},1\leq i\leq n\right)$ of all the inliers can be obtained using

(3) $$\begin{array}{l} C_{2D}=\frac{\sum_{i=1}^{n}x_{i}}{n}\\ C_{3D}=\frac{\sum_{i=1}^{n}X_{i}}{n} \end{array}$$

The origins of the 2D and 3D points of the inliers can be transformed to the centroids as follows:

(4) $$\begin{array}{l} x_{i}=x_{i}-C_{2D}\\ X_{i}=X_{i}-C_{3D} \end{array}$$

To normalize the inliers, the average distance from the inliers to the origins is set to $\sqrt{2}$ and $\sqrt{3}$ for the 2D and 3D points, respectively, using the following formulas:

(5) $$\begin{array}{l} x_{i}^{nor}=\frac{\sqrt{2}n}{\sum_{j=1}^{n}\left\|x_{j}\right\|}x_{i}\\ X_{i}^{nor}=\frac{\sqrt{3}n}{\sum_{j=1}^{n}\left\|X_{j}\right\|}X_{i} \end{array}$$

where $x_{i}^{nor}$ and $X_{i}^{nor}$ are the new point correspondences after normalization for pose estimation, and we can obtain the transformation from the original inliers to the new inliers using the following formula:

(6) $$\begin{array}{l} \left[\begin{array}{c} x_{i}^{nor}\\ 1 \end{array}\right]=T_{x}\left[\begin{array}{c} x_{i}\\ 1 \end{array}\right]\\ \left[\begin{array}{c} X_{i}^{nor}\\ 1 \end{array}\right]=T_{X}\left[\begin{array}{c} X_{i}\\ 1 \end{array}\right] \end{array}$$

where

(7) $$\begin{array}{l} T_{x}=\left[\begin{array}{ccc} \frac{\sqrt{2}n}{\sum_{j=1}^{n}\left\|x_{j}\right\|} & 0 & -\frac{\sqrt{2}n}{\sum_{j=1}^{n}\left\|x_{j}\right\|}\cdot C_{2D}\left(1\right)\\ 0 & \frac{\sqrt{2}n}{\sum_{j=1}^{n}\left\|x_{j}\right\|} & -\frac{\sqrt{2}n}{\sum_{j=1}^{n}\left\|x_{j}\right\|}\cdot C_{2D}\left(2\right)\\ 0 & 0 & 1 \end{array}\right] \end{array}\;T_{X}=\left[\begin{array}{cccc} \frac{\sqrt{3}n}{\sum_{j=1}^{n}\left\|X_{j}\right\|} & 0 & 0 & -\frac{\sqrt{3}n}{\sum_{j=1}^{n}\left\|X_{j}\right\|}\cdot C_{3D}\left(1\right)\\ 0 & \frac{\sqrt{3}n}{\sum_{j=1}^{n}\left\|X_{j}\right\|} & 0 & -\frac{\sqrt{3}n}{\sum_{j=1}^{n}\left\|X_{j}\right\|}\cdot C_{3D}\left(2\right)\\ 0 & 0 & \frac{\sqrt{3}n}{\sum_{j=1}^{n}\left\|X_{j}\right\|} & -\frac{\sqrt{3}n}{\sum_{j=1}^{n}\left\|X_{j}\right\|}\cdot C_{3D}\left(3\right)\\ 0 & 0 & 0 & 1 \end{array}\right]$$

In the next section, $x_{i}^{nor}$ and $X_{i}^{nor}$ are used to estimate the absolute pose.

#### 2.2.3. Absolute Pose Estimation and Refining

Here, the first stage is to estimate the coarse pose, and the second stage is to refine the absolute pose.

In this section, we use all new inliers $x_{i}^{nor}$ and $X_{i}^{nor}$ to estimate the coarse pose with the DLT algorithm. According to a standard pinhole camera, the projection can be written as

(8) $$z_{c}\left[\begin{array}{c} x_{i}^{nor}\\ 1 \end{array}\right]=K\cdot \left[\begin{array}{cc} R & t \end{array}\right]\left[\begin{array}{c} X_{i}^{nor}\\ 1 \end{array}\right],$$

where *K* is the intrinsic parameter matrix, which is expressed as

(9) $$K=\left[\begin{array}{ccc} f & 0 & u_{0}\\ 0 & f & v_{0}\\ 0 & 0 & 1 \end{array}\right],$$

where *f* is the focal length in pixels, and $\left(\begin{array}{cc} u_{0} & v_{0} \end{array}\right)$ is the principal point, i.e., the center of the image in this paper. Now, a matrix *P* can be obtained from Equation (8) by eliminating the unknown parameter *z_c_*.

(10) $$P=\left[\begin{array}{cccccccccccc} x_{1} & y_{1} & z_{1} & 0 & 0 & 0 & -u_{1}x_{1} & -u_{1}y_{1} & -u_{1}z_{1} & 1 & 0 & -u_{1}\\ 0 & 0 & 0 & x_{1} & y_{1} & z_{1} & -v_{1}x_{1} & -v_{1}y_{1} & -v_{1}z_{1} & 0 & 1 & v_{1}\\ \vdots & \vdots & \vdots & \vdots & \vdots & \vdots & \vdots & \vdots & \vdots & \vdots & \vdots & \vdots \\ \vdots & \vdots & \vdots & \vdots & \vdots & \vdots & \vdots & \vdots & \vdots & \vdots & \vdots & \vdots \\ x_{n} & y_{n} & z_{n} & 0 & 0 & 0 & -u_{n}x_{n} & -u_{n}y_{n} & -u_{n}z_{n} & 1 & 0 & -u_{n}\\ 0 & 0 & 0 & x_{n} & y_{n} & z_{n} & -v_{n}x_{n} & -v_{n}y_{n} & -v_{n}z_{n} & 0 & 1 & -v_{n} \end{array}\right]$$

where the size of the matrix *P* is 2*n* × 12, and *n* is the number of the inliers. Then, singular value decomposition is carried out for the matrix $P^{T}P$.

(11) $$\left[\begin{array}{ccc} U & S & V \end{array}\right]=\mathrm{SVD}\left(P^{T}P\right).$$

According to the least squares solution of the homogeneous equation system, we can obtain the rotation matrix $R^{nor}$ and translation vector $t^{nor}$ after normalization.

(12) $$\left[\begin{array}{cc} R^{nor} & t^{nor} \end{array}\right]=\left[\begin{array}{cccc} V\left(1,12\right) & V\left(2,12\right) & V\left(3,12\right) & V\left(4,12\right)\\ V\left(5,12\right) & V\left(6,12\right) & V\left(7,12\right) & V\left(8,12\right)\\ V\left(9,12\right) & V\left(10,12\right) & V\left(11,12\right) & V\left(12,12\right) \end{array}\right].$$

Lastly, the original rotation matrix and translation vector can be given using

(13) $$\left[\begin{array}{cc} R^{ori} & t^{ori} \end{array}\right]=T_{x}^{-1}\cdot \left[\begin{array}{cc} R^{nor} & t^{nor} \end{array}\right]\cdot T_{X}.$$

Having completed the coarse pose estimation, it is subsequently refined. The procedure is formulated as the optimization of a cost function. Consequently, the first stage is to develop a cost function. In this paper, the optimization of the cost function is to minimize the reprojection error.

(14) $$\left\{\begin{array}{l} F\left(R^{ori},t^{ori}\right)=\underset{R^{ori},t^{ori}}{\min}\sum_{i=1}^{n}\left\|x_{i}-x_{i}^{’}\right\|\\ z_{c\_i}\left[\begin{array}{c} x_{i}^{’}\\ 1 \end{array}\right]=K\cdot \left[\begin{array}{cc} R^{ori} & t^{ori} \end{array}\right]\left[\begin{array}{c} X_{i}\\ 1 \end{array}\right] \end{array}\right..$$

**Figure 3:**
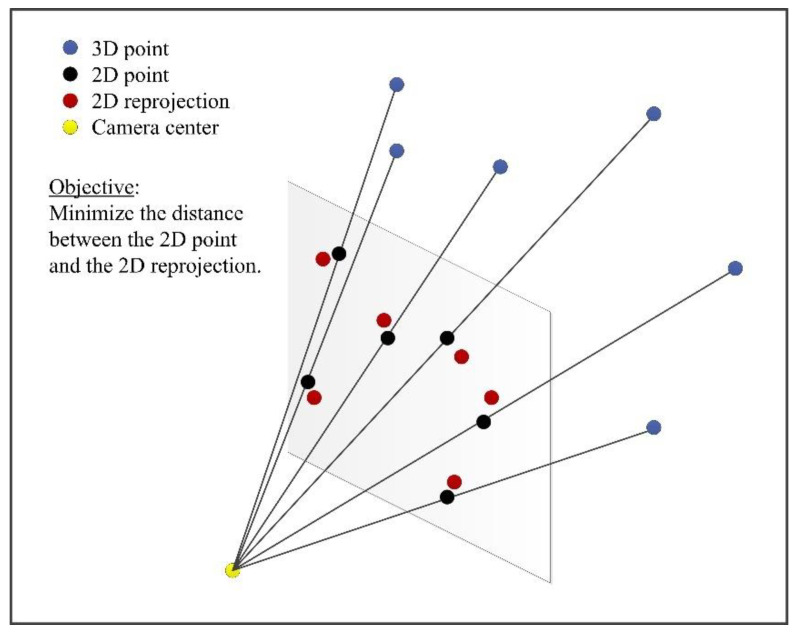
The reprojection error.

Next, the pose is refined to solve the cost function $F\left(R^{ori},t^{ori}\right)$. In this paper, we refine the pose by minimizing the reprojection error using the error transferring model and gradient descent to obtain the optimal solution from Equation (14). The process for minimizing the reprojection error is illustrated in Figure 3.

The pose refinement process described in Equation (14) is applied to obtain the optimal solution of $R^{ori}$ and $t^{ori}$; however, the pose parameters are rotation angle $O^{ori}$ and translation vector $t^{ori}$, not $R^{ori}$ and $t^{ori}$. $R^{ori}$ is the rotation matrix based on the rotation angles $O^{ori}$. Hence, the goal of pose refining is to minimize the reprojection error using rotation angles $O^{ori}$ and translation vector $t^{ori}$. If the optimization parameters are $R^{ori}$ and $t^{ori}$, we can minimize the reprojection error, and the optimal solution of $R^{ori}$ and $t^{ori}$ can be obtained. The problem is that components of the rotation matrix $R^{ori}$ are not theoretically independent because $R^{ori}$ has nine parameters with three degrees of freedom (dof), whereas they are independent if the optimization parameters are $R^{ori}$ and $t^{ori}$. Then, the computed result might not be a rotation matrix. Actually, the rotation matrix is orthogonal, but we cannot obtain an orthogonal matrix if $R^{ori}$ is directly used as the optimization parameter because of the lack of constraints.

To solve this problem, the rotation matrix $R^{ori}$ is represented by rigid body transformation using rotation angles $O^{ori}\left(\begin{array}{ccc} \alpha & \beta & \gamma \end{array}\right)$ as follows:

(15) $$\begin{array}{l} R^{ori}=f_{rod}\left(O^{ori}\right)\\ f_{rod}\left(O^{ori}\right)=\left[\begin{array}{ccc} \cos\beta \cos\gamma -\sin\alpha \sin\beta \sin\gamma & \cos\alpha \sin\gamma & \sin\beta \cos\gamma +\cos\beta \sin\alpha \sin\gamma \\ -\cos\beta \sin\gamma -\sin\beta \sin\alpha \cos\gamma & \cos\alpha \cos\gamma & -\sin\beta \sin\gamma +\cos\beta \sin\alpha \cos\gamma \\ -\sin\beta \cos\alpha & -\sin\alpha & \cos\beta \cos\alpha \end{array}\right] \end{array}$$

Then, the cost function is rewritten as

(16) $$F\left(O^{ori},t^{ori}\right)=\underset{O^{ori},t^{ori}}{\min}\sum_{i=1}^{n}\left\|x_{i}-x_{i}^{’}\right\|.$$

**Figure 4:**
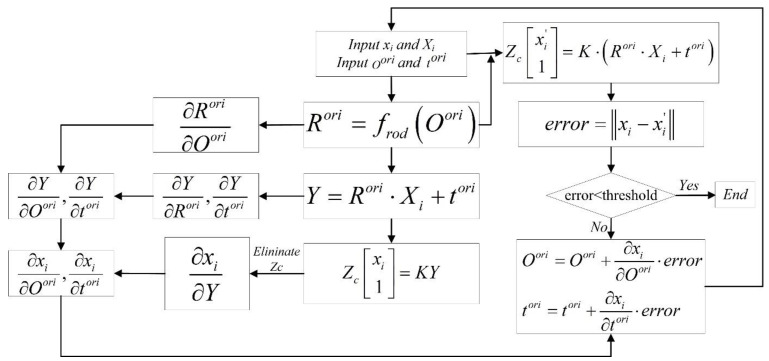
Pose refining.

Using the error transferring model and gradient descent, the reprojection error $\left\|x_{i}-x_{i}^{’}\right\|$ and the partial derivative with respect to the rotation angle and translation vector need to be obtained. Hence, the procedure of pose refining is illustrated in Figure 4.

Absolute pose refining can be achieved using the procedure illustrated in Figure 4, which always results in a valid rotation matrix and translation vector.

**Figure 5:**
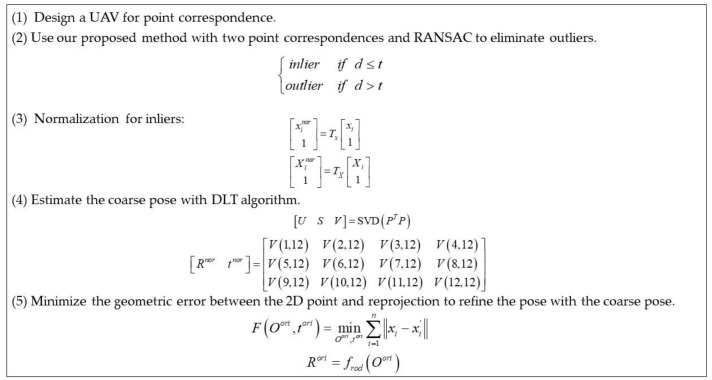
Brief calculating process of our proposed method.

In brief, we proposed an accurate and robust method for camera pose estimation with a UAV using RANSAC, which is outlined below (Figure 5).

## 3. Experiments and Results

In this section, our proposed method is thoroughly and directly tested using synthetic data in terms of robustness to camera position noise, numerical stability, performance analysis of outlier ratio, noise sensitivity, and computational speed, and it is compared with P3P [18], RPnP (robust O (n) solution to the perspective-n-point) [41], and EPnP (efficient solution to the perspective-n-point) [42] solvers. Then, we manufactured a prototype of the UAV to obtain real images, and our proposed method was indirectly tested using real images to verify the feasibility in practice.

### 3.1. Synthetic Data

A virtual perspective camera was synthesized with a resolution of 1280 × 800, pixel size of 14 μm × 14 μm, and focal length of 50 mm. In our proposed method, the camera position is known; hence, the virtual camera was fixed at $\left(\begin{array}{ccc} 0 & 0 & 0 \end{array}\right)$ in the world frame.

A total of 400 3D points in the world frame were randomly selected in the box of [–17, 17] × [−11, 11] × [100, 110]. Then, 400 2D points (i.e., the projections of the 3D points) were obtained through the virtual perspective camera. We then used the 2D–3D point correspondences to thoroughly test our proposed method with RANSAC. The minimal subset was set to a size of two, three, five, and five for our proposed method, P3P, RPnP, and EPnP, respectively. Depending on the testing needs, some outliers were added to the point correspondences in this section.

#### 3.1.1. Robustness to Camera Position Noise

Compared with the existing methods (P3P, RPnP, and EPnP solvers), the difference of our proposed method is the use of the camera position as prior knowledge. In practice, the camera position obtained using RTK or the total station is not absolutely accurate. Hence, to eliminate the impact of the camera position noise when testing the performance, it was essential to analyze the effect of camera position noise on the absolute pose estimation with RANSAC.

**Figure 6:**
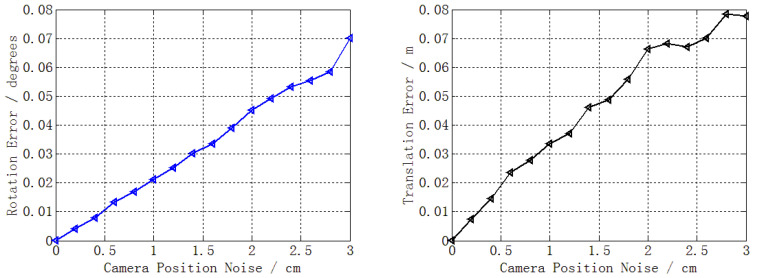
Rotation error (**left**) and translation error (**right**).

The camera position is generally given by RTK or the total station, whose accuracy is better than 3 cm or 0.5 cm, respectively. Hence, we added zero-mean Gaussian noise to the camera position, varying the noise deviation level from 0 to 3 cm [50,51]. In addition, the outlier ratio was set to 30% as suggested in [1]. A total of 50,000 independent trials were performed at each noise level. The mean errors of rotation and translation at each noise level are reported in Figure 6.

Figure 6 shows that, as the camera position noise increased, so did the rotation error and translation error. When the noise was 3 cm, the maximum errors were 0.07° for rotation and 0.08 m for translation. The maximum error revealed that the performance degradation caused by the camera position noise was very slight.

#### 3.1.2. Numerical Stability

**Figure 7:**
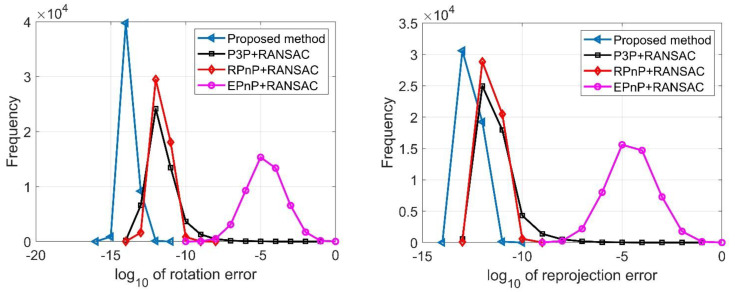
Numerical stability of rotation (**left**) and reprojection (**right**) for our proposed method (blue), P3P + RANSAC (black), RPnP + RANSAC (red), and EPnP + RANSAC (purple).

In this section, no noise was added to the 2D–3D point correspondences and camera position, and the outlier ratio was 30%. A total of 50,000 trials were performed independently for our proposed method, P3P + RANSAC, RPnP + RANSAC, and EPnP + RANSAC. Then, the numerical stability of rotation and reprojection was determined, as reported in Figure 7.

Figure 7 presents the distributions of rotation error (left) and reprojection error (right). We can see that our proposed method had the highest numerical stability among the methods according to the distribution of the log_10_ value of rotation error and reprojection error. P3P + RANSAC and RPnP + RANSAC performed similarly with the second best numerical stability, while EPnP + RANSAC had the worst numerical stability.

#### 3.1.3. Performance Analysis of Outlier Ratio

**Figure 8:**
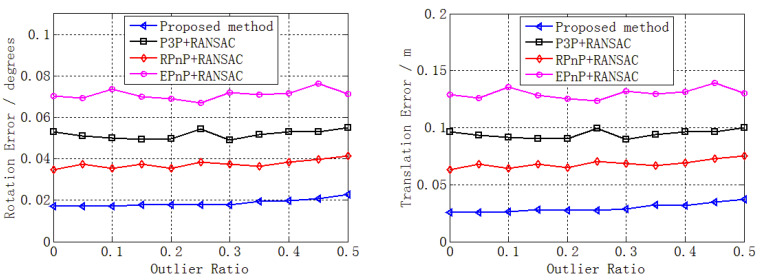

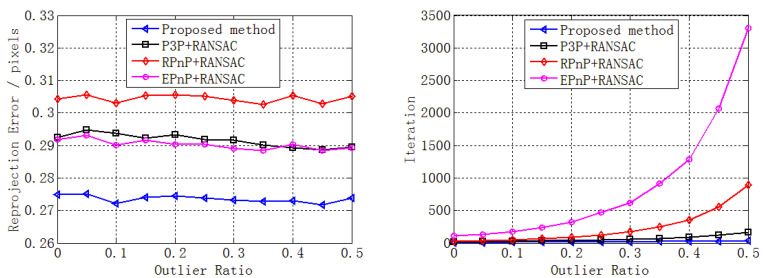
Performance analysis of outlier ratio in terms of rotation error (**top left**), translation error (**top right**), reprojection error (**bottom left**), and number of iterations (**bottom right**) for our proposed method (blue), P3P + RANSAC (black), RPnP + RANSAC (red), and EPnP + RANSAC (purple).

We added very small noise (0.01 pixels) to the 2D point and performed a performance analysis of the outlier ratio. A total of 50,000 independent trials were performed at each outlier ratio level. Then, the mean rotation error, translation error, reprojection error, and number of iterations with different outlier ratios (0–0.5) were determined, as reported in Figure 8.

Figure 8 shows that the errors changed slightly when the outlier ratio increased from 0 to 0.5. The reason is mainly that many inliers could still be obtained through RANSAC despite the existence of mass outliers. In addition, these inliers were enough to estimate the pose without noise. Accordingly, the errors changed slightly, highlighting the numerical stability, which is consistent with the results in Section 3.1.2. However, Figure 6 (bottom right) shows that the number of iterations increased sharply, especially for RPnP + RANSAC and EPnP + RANSAC. This was caused by the increase in outlier ratio, consistent with Equation (2); the main reason was that the size of the minimal subset was larger than that of our method and the P3P solver.

Despite the errors changing slightly when the outlier ratio increased, our proposed method still performed better than the other three solvers, as shown in Figure 8. In addition, the performance superiority of our proposed method in terms of the number of iterations improved sharply when the outlier ratio increased compared with the other three methods, especially with respect to RPnP + RANSAC and EPnP + RANSAC.

#### 3.1.4. Noise Sensitivity

**Figure 9:**
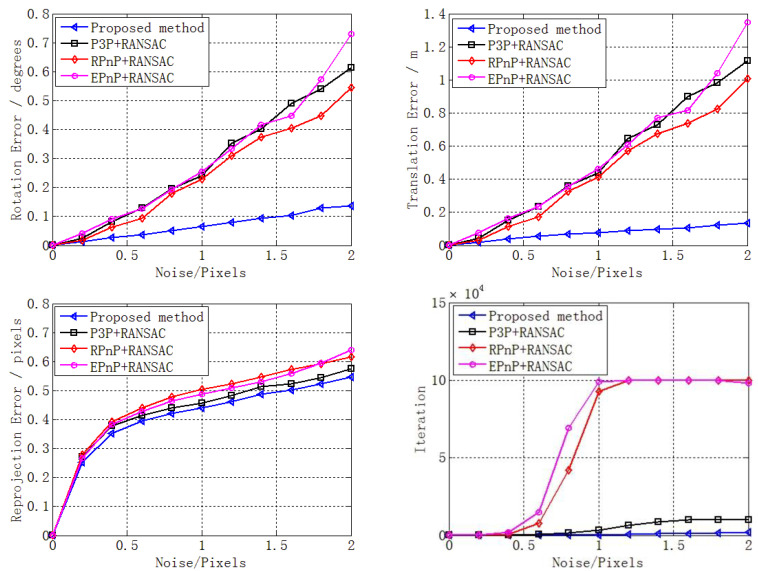
Noise sensitivity in terms of rotation error (**top left**), translation error (**top right**), reprojection error (**bottom left**), and iteration (**bottom right**) for our proposed method (blue), P3P + RANSAC (black), RPnP + RANSAC (red), and EPnP + RANSAC (purple).

There are two sources of noise in the inliers (i.e., 2D point noise and 3D point noise). They are not independent because the 2D point is the image projection of the 3D point. Consequently, the noise of the 3D point is reflected in the noise of the 2D point. Hence, zero-mean Gaussian noise was only added to the 2D points, with the noise deviation level varying from 0 to 2 pixels in this section. A total of 50,000 independent trials were performed at each noise level, and then the mean errors of rotation, translation, and reprojection, as well as the mean number of iterations, at each noise level are reported in Figure 9.

Figure 9 shows that, as the noise increased, the errors of rotation, translation, and reprojection increased for all the four methods. In addition, our proposed method performed much better than the other three solvers in terms of rotation error, translation error, and number of iterations, while the performance superiority of our proposed method increased sharply when the noise increased compared with the other three methods. In addition, our proposed method performed slightly better than the other three solvers in terms of reprojection error. In brief, this section highlighted the excellent noise sensitivity of our proposed method.

#### 3.1.5. Computational Speed

We analyzed the computational speed under different noise and outlier ratio levels on a 3.3 GHz four-core laptop.

**Figure 10:**
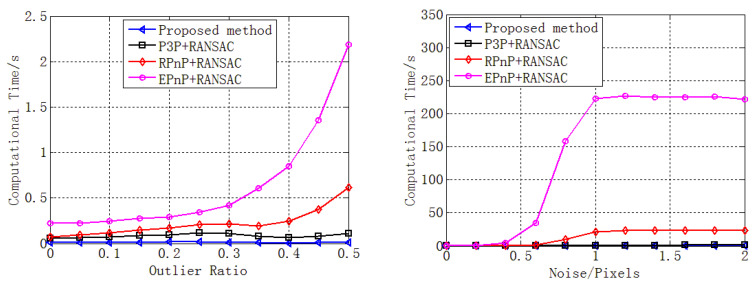
Computational time under different noise (**left**) and outlier ratio (**right**) levels for our proposed method (blue), P3P + RANSAC (black), RPnP + RANSAC (red), and EPnP + RANSAC (purple).

First, the noise was maintained at 0.5 pixels while the outlier ratio was varied from 0 to 0.5. Then, 50,000 independent trials were performed at each outlier ratio level. The mean computational time was determined, and the results are reported in Figure 10 (left).

Then, the outlier ratio was maintained at 0.3 while the noise was varied from 0 to 2 pixels. Then, 50,000 independent trials were performed at each noise level. The mean computational time was determined, and the results are reported in Figure 10 (right).

Figure 10 shows that, as the outlier ratio or noise increased, so did the computational time of all the four methods. This is consistent with the analysis of the number of iterations in Section 3.1.3 and Section 3.1.4. The increase in computational time was mainly caused by the increase in the number of iterations, as shown in Figure 8 (bottom right) and Figure 9 (bottom right).

Moreover, the computational speed of our proposed method was much faster than that of RPnP + RANSAC and EPnP + RANSAC, and the performance superiority of our proposed method increased sharply when the outlier ratio or noise increased.

### 3.2. Real Images

**Figure 11:**
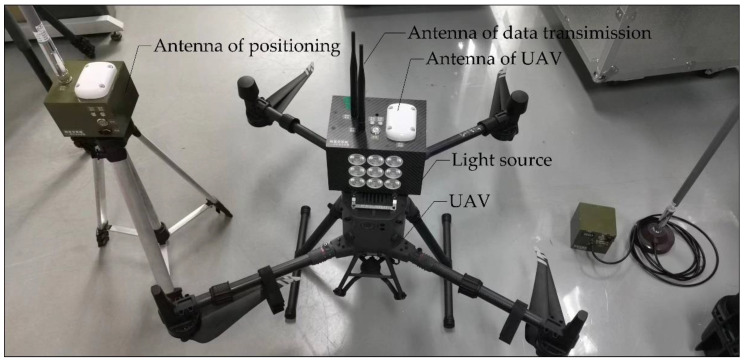
Prototype of the UAV featuring the mounted light source, used as the 3D point.

To obtain 2D–3D point correspondences from real images, we manufactured a prototype of a UAV (DJI M300 PRO made by DJI in Shenzhen, China) using RTK (Sino GNSS, K705 made by South Group in Guangzhou, China) according to the design in Section 2.1, as shown in Figure 11.

**Figure 12:**
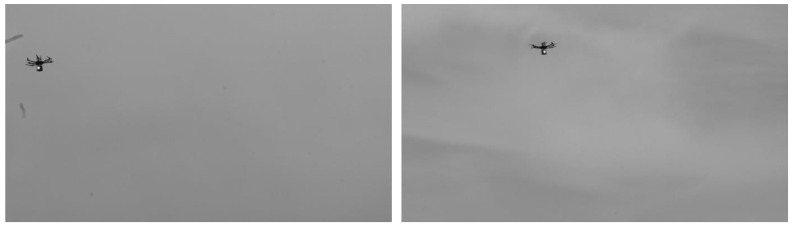
Real images captured in the pure sky by the left camera (**left**) and right camera (**right**). A total of 40 images were captured using the light source on the UAV as a 3D point.

Due to the limited endurance of our prototype UAV, we were only able to capture approximately 40 real images in the experiment before the UAV ran out of power and landed. These real images of the light source on the UAV were captured using two cameras, as shown in Figure 12. The two cameras were located at [−100, −100, 0] and [100, −100, 0] m in the world frame, and their focal lengths were both 50 mm. The two cameras both pointed to [0, 0, 20]. The UAV flew in the box of [−15, 15] × [−15, 15] × [5, 35] m in the world frame.

The FOV of the cameras was the pure sky; hence, no natural feature was used as 3D point. However, we used the light source on the UAV as the 3D point for camera pose estimation. The 3D point of the light source in the world frame was given by RTK, and the corresponding 2D point in the real image was obtained by feature extraction. Then, 40 2D–3D point correspondences were obtained for pose estimation, consisting of inliers and outliers.

In real scenarios, the ground truth of the camera pose is unknown; hence, we could not directly test the performance of our proposed method for rotation or translation. However, the ground truth of the 3D point could be obtained through RTK, which could be indirectly used to test our proposed method. First, we estimated the pose of the two cameras using the 40 2D–3D point correspondences with RANSAC. Then, another 10 2D–3D point correspondences were obtained using the UAV. Note that these 10 correspondences had to be valid with no outliers; hence, the UAV kept flying until all 10 correspondences were inliers. Lastly, stereo vision using the two cameras with the pose estimated by our proposed method and the other three methods was employed to calculate the positions of the 10 3D points, and the mean relative error of the position between the ground truth given by RTK and the measured value given by stereo vision [52], as well as the mean reprojection error, was calculated, as reported in Table 1.

Because the error of pose estimation can affect the measurement accuracy of stereo vision, the measurement error of stereo vision can reflect the performance of pose estimation. In addition, the measurement accuracy of stereo vision can affect the reprojection accuracy; hence, the reprojection error of stereo vision can also reflect the performance of pose estimation. Consequently, the position relative error and reprojection error could be used to indirectly test the performance of our proposed method.

From Table 1, it can be seen that our proposed method performed better than the other three methods according to the position relative error and reprojection error. Our proposed method achieved the best results, whereas EPnP + RANSAC performed the worst. This is consistent with the results from the synthetic data, highlighting that our proposed method could work well with both synthetic data and real images.

In addition, even though only 40 real images (in contrast to the 400 synthetic data) were used in the experiment, we still achieved good experimental results, and our method performed better than the other methods. Actually, because more point correspondences lead to better accuracy, we can expect better results with more real images, which was also reflected in the synthetic data.

## 4. Discussion

Pose estimation is one of the key steps in computer vision, photogrammetry, SLAM, and SfM (structure from motion). In this paper, we proposed an accurate and robust method for absolute pose estimation with UAV using RANSAC. Because obtaining artificial 3D control points with high accuracy is time-consuming and a small point set can lead to low measuring accuracy, we designed a customized UAV to efficiently obtain mass 3D points. A light source was mounted on the UAV and used as 3D points. The position of the 3D point was given by RTK mounted on the UAV, while the position of the corresponding 2D point was obtained through feature extraction. The 2D–3D point correspondences presented some outliers because of the failure of feature extraction, RTK error, and wrong matches of 2D and 3D points. Hence, RANSAC was used to remove the outliers and obtain the coarse pose. Then, we proposed a method to refine the coarse pose. This is unlike existing pose estimation solvers, and the advantages of our proposed method are discussed below.

### 4.1. Differences and Advantages

The first difference of the method lies in the approach to obtaining the 2D–3D point correspondences. Existing methods generally use the feature points in the environment of the FOV. The 2D–3D point correspondences can be obtained by feature extraction and matching. In addition, some methods use several artificial 3D control points and obtain their positions using the total station or RTK. In this paper, we designed a UAV with a light source and RTK to efficiently obtain the 2D–3D point correspondences. Mass 2D–3D point correspondences can be obtained even if there is no feature point or artificial 3D control point. This approach can be extended to the application scenarios of pose estimation, thereby improving the efficiency of obtaining point correspondences.

Because the size of the minimal subset of 2D–3D point correspondences is two in our proposed method, which is smaller than the other methods, the iterations are smaller. This is the main reason why our proposed method was faster than the other methods, as described in Section 3.1.5. In addition, because our method without RANSAC has higher accuracy [35], it can save time when we refine the coarse pose. This is another reason why our proposed method was faster than the other methods.

Although our proposed method uses camera position as prior knowledge unlike other methods, it does not cause a loss of accuracy, as described in Section 3.1.1. This is a reason why our proposed method had improved numerical stability.

Then, we use RANSAC to remove the outliers, which makes our method robust and allows obtaining the coarse pose estimation. We developed a cost function in reprojection error to refine the coarse pose estimation through gradient descent, making our method accurate.

Lastly, because of the higher accuracy of pose estimation, a smaller position error and reprojection error can be obtained using stereo vision with our proposed method, thereby verifying the feasibility for pose estimation in practical application.

### 4.2. Future Work

In the future, we will optimize the mechanical structure of the prototype to improve the accuracy of RTK during the UAV flight and reduce the outliers. This can improve the computational speed. In addition, because the prototype mounts a light source and RTK on the UAV, this brings extra load and results in a shorter flight time. The flight time determines the size of the point correspondences. Hence, if we want more point correspondences, the power supply system of the prototype will need to be optimized.

## 5. Conclusions

We designed and manufactured a prototype UAV to obtain mass 2D–3D point correspondences for camera pose estimation. We mounted a light source on the UAV and used it as 3D point, whose position was given by the RTK mounted on the UAV. Because the 2D–3D point correspondences had outliers, an accurate and robust method using RANSAC was proposed in this paper to estimate the coarse pose. Then, we used a cost function in reprojection error to refine the coarse pose through gradient descent.

The experimental results showed that our proposed method works well using synthetic data and real scenarios. It is particularly suitable for estimating camera pose in cases where there is no feature point or artificial 3D control point in the environment. Our proposed method can extend the application scenarios of pose estimation and improve the efficiency of obtaining point correspondences.

## Figures and Tables

**Table 1:** Position relative error and reprojection error in real images.

Method	Proposed Method	P3P + RANSAC	EPnP + RANSAC	RPnP + RANSAC
Position relative error	0.08%	0.14%	0.19%	0.13%
Reprojection error/pixel	0.28	0.49	0.61	0.46

## Data Availability

The data presented in this study are available in the manuscript.

## References

[1] Vongkulbhisal J., De la Torre F., Costeira J.P. (2018). Discriminative optimization: Theory and applications to computer vision. IEEE Trans. Pattern Anal. Mach. Intell..

[2] Zhou F., Cui Y., Wang Y., Liu L., Gao H. (2013). Accurate and robust estimation of camera parameters using RANSAC. Opt. Lasers Eng..

[3] Lourakis M., Terzakis G. A globally optimal method for the PnP problem with MRP rotation parameterization. Proceedings of the International Conference on Pattern Recognition.

[4] Hartley R., Zisserman A. (2003). Multiple View Geometry in Computer Vision.

[5] Brachmann E., Krull A., Nowozin S., Shotton J., Michel F., Gumhold S., Rother C. Dsac-differentiable ransac for camera localization. Proceedings of the IEEE Conference on Computer Vision and Pattern Recognition.

[6] Guan B., Zhao J., Li Z., Sun F., Fraundorfer F. Minimal solutions for relative pose with a single affine correspondence. Proceedings of the IEEE Conference on Computer Vision and Pattern Recognition.

[7] Zhou L., Ye J., Kaess M. A stable algebraic camera pose estimation for minimal configurations of 2D/3D point and line correspondences. Proceedings of the Asian Conference on Computer Vision.

[8] Zheng Y., Sugimoto S., Sato I., Okutomi M. A general and simple method for camera pose and focal length determination. Proceedings of the IEEE Conference on Computer Vision and Pattern Recognition.

[9] Gao X.S., Hou X.R., Tang J., Cheng H.F. (2003). Complete solution classification for the perspective-three-point problem. IEEE Trans. Pattern Anal. Mach. Intell..

[10] Lacey A.J., Pinitkarn N., Thacker N.A. An Evaluation of the Performance of RANSAC Algorithms for Stereo Camera Calibrarion. Proceedings of the Eleventh British Machine Vision Conference, University of Bristol.

[11] Heikkila J. (2000). Geometric camera calibration using circular control points. IEEE Trans. Pattern Anal. Mach. Intell..

[12] Gong X., Lv Y., Xu X., Wang Y., Li M. (2021). Pose Estimation of Omnidirectional Camera with Improved EPnP Algorithm. Sensors.

[13] Hu Y., Fua P., Wang W., Salzmann M. Single-stage 6d object pose estimation. Proceedings of the IEEE Conference on Computer Vision and Pattern Recognition.

[14] Guo K., Ye H., Chen H., Gao X. (2022). A New Method for Absolute Pose Estimation with Unknown Focal Length and Radial Distortion. Sensors.

[15] Hadfield S., Lebeda K., Bowden R. (2018). HARD-PnP: PnP optimization using a hybrid approximate representation. IEEE Trans. Pattern Anal. Mach. Intell..

[16] Zheng Y., Kuang Y., Sugimoto S., Astrom K., Okutomi M. Revisiting the pnp problem: A fast, general and optimal solution. Proceedings of the IEEE International Conference on Computer Vision.

[17] Wu Y., Hu Z. (2006). PnP problem revisited. J. Math. Imaging Vis..

[18] Kneip L., Scaramuzza D., Siegwart R. A novel parametrization of the perspective-three-point problem for a direct computation of absolute camera position and orientation. Proceedings of the IEEE Conference on Computer Vision and Pattern Recognition.

[19] Wolfe W.J., Mathis D., Sklair C.W., Magee M. (1991). The perspective view of three points. IEEE Comput. Archit. Lett..

[20] Kanaeva E., Gurevich L., Vakhitov A. Camera pose and focal length estimation using regularized distance constraints. Proceedings of the British Machine Vision Conference.

[21] Yin X., Ma L., Tan X., Qin D. (2021). A Robust Visual Localization Method with Unknown Focal Length Camera. IEEE Access.

[22] Penate-Sanchez A., Andrade-Cetto J., Moreno-Noguer F. (2013). Exhaustive linearization for robust camera pose and focal length estimation. IEEE Trans. Pattern Anal. Mach. Intell..

[23] Nakano G. A versatile approach for solving PnP, PnPf, and PnPfr problems. Proceedings of the European Conference on Computer Vision.

[24] Josephson K., Byrod M. Pose estimation with radial distortion and unknown focal length. Proceedings of the IEEE Conference on Computer Vision and Pattern Recognition.

[25] Kukelova Z., Bujnak M., Pajdla T. Real-time solution to the absolute pose problem with unknown radial distortion and focal length. Proceedings of the IEEE International Conference on Computer Vision.

[26] Triggs B. Camera pose and calibration from 4 or 5 known 3d points. Proceedings of the Seventh IEEE International Conference on Computer Vision.

[27] Wu Y., Li Y., Hu Z. (2008). Detecting and handling unreliable points for camera parameter estimation. Int. J. Comput. Vis..

[28] Zhao Z., Ye D., Zhang X., Chen G., Zhang B. (2016). Improved direct linear transformation for parameter decoupling in camera calibration. Algorithms.

[29] Barone F., Marrazzo M., Oton C.J. (2020). Camera calibration with weighted direct linear transformation and anisotropic uncertainties of image control points. Sensors.

[30] Kukelova Z., Bujnak M., Pajdla T. Closed-form solutions to minimal absolute pose problems with known vertical direction. Proceedings of the Asian Conference on Computer Vision.

[31] Sweeney C., Flynn J., Nuernberger B., Turk M., Höllerer T. Efficient computation of absolute pose for gravity-aware augmented reality. Proceedings of the IEEE International Symposium on Mixed and Augmented Reality.

[32] Bujnák M. (2012). Algebraic Solutions to Absolute Pose Problems. Ph.D. Thesis.

[33] D’Alfonso L., Garone E., Muraca P., Pugliese P. On the use of IMUs in the PnP Problem. Proceedings of the International Conference on Robotics and Automation.

[34] Kalantari M., Hashemi A., Jung F., Guédon J.P. (2011). A new solution to the relative orientation problem using only 3 points and the vertical direction. J. Math. Imaging Vis..

[35] Guo K., Ye H., Zhao Z., Gu J. (2021). An efficient closed form solution to the absolute orientation problem for camera with unknown focal length. Sensors.

[36] Derpanis K.G. (2010). Overview of the RANSAC Algorithm. Image Rochester NY.

[37] Fischler M.A., Bolles R.C. (1981). Random sample consensus: A paradigm for model fitting with applications to image analysis and automated cartography. Commun. ACM.

[38] Botterill T., Mills S., Green R. Fast RANSAC hypothesis generation for essential matrix estimation. Proceedings of the International Conference on Digital Image Computing: Techniques and Applications.

[39] Ferraz L., Binefa X., Moreno-Noguer F. Very fast solution to the PnP problem with algebraic outlier rejection. Proceedings of the IEEE Conference on Computer Vision and Pattern Recognition.

[40] Zakharov S., Shugurov I., Ilic S. Dpod: 6d pose object detector and refiner. Proceedings of the IEEE/CVF International Conference on Computer Vision.

[41] Li S., Xu C., Xie M. (2012). A robust O (n) solution to the perspective-n-point problem. IEEE Trans. Pattern Anal. Mach. Intell..

[42] Lepetit V., Moreno-Noguer F., Fua P. (2009). Epnp: An accurate o (n) solution to the pnp problem. Int. J. Comput. Vis..

[43] Ding Y., Yang J., Ponce J., Kong H. Minimal solutions to relative pose estimation from two views sharing a common direction with unknown focal length. Proceedings of the IEEE/CVF Conference on Computer Vision and Pattern Recognition.

[44] Liu L., Li H., Dai Y. Efficient global 2d-3d matching for camera localization in a large-scale 3d map. Proceedings of the IEEE International Conference on Computer Vision.

[45] Germain H., Bourmaud G., Lepetit V. Sparse-to-dense hypercolumn matching for long-term visual localization. Proceedings of the International Conference on 3D Vision.

[46] Jiang Y., Xu Y., Liu Y. (2013). Performance evaluation of feature detection and matching in stereo visual odometry. Neurocomputing.

[47] Dang Z., Yi K.M., Hu Y., Wang F., Fua P., Salzmann M. Eigendecomposition-free training of deep networks with zero eigenvalue-based losses. Proceedings of the European Conference on Computer Vision.

[48] Botterill T., Mills S., Green R. Refining essential matrix estimates from RANSAC. Proceedings of the Image and Vision Computing New Zealand.

[49] Torr P.H., Murray D.W. (1997). The development and comparison of robust methods for estimating the fundamental matrix. Int. J. Comput. Vis..

[50] Guo K., Ye H., Gu J., Chen H. (2021). A novel method for intrinsic and extrinsic parameters estimation by solving perspective-three-point problem with known camera position. Appl. Sci..

[51] Forlani G., Dall’Asta E., Diotri F., Cella U.M.D., Roncella R., Santise M. (2018). Quality assessment of DSMs produced from UAV flights georeferenced with on-board RTK positioning. Remote Sens..

[52] Lazaros N., Sirakoulis G.C., Gasteratos A. (2008). Review of stereo vision algorithms: From software to hardware. Int. J. Optomechatronics.

